# Convalescent plasma to treat critically ill patients with COVID-19: framing the need for randomised clinical trials

**DOI:** 10.1186/s13054-020-03163-3

**Published:** 2020-07-20

**Authors:** Manu Shankar-Hari, Lise Estcourt, Heli Harvala, David Roberts, David K. Menon, On behalf of the United Kingdom SARS-CoV-2 Convalescent Plasma Evaluation (SCoPE) Consortium, Manu Shankar-Hari, Lise Estcourt, Heli Harvala, David Roberts, David K. Menon

**Affiliations:** 1 School of Immunology & Microbial Sciences, Kings College London, 5th Floor, Southwark Wing, London, SE1 9RT UK; 2 Guy’s and St Thomas’ NHS Foundation Trust, ICU Support Offices, 1st Floor, East Wing, St Thomas’ Hospital, London, SE1 7EH UK; 3 NHS Blood and Transplant, Level 2, John Radcliffe Hospital, Oxford, OX3 9BQ UK; 4 Radcliffe Department of Medicine, University of Oxford, Oxford, UK; 5 National Microbiology Services, NHS Blood and Transplant, Charcot Road, Colindale, NW9 5BG UK; 6 Division of Infection and Immunity, University College of London, London, WC1E 6JF UK; 7 Division of Anaesthesia, Department of Medicine, University of Cambridge, Box 93, Addenbrooke’s Hospital, Cambridge, CB2 0NUI UK

We are in a severe acute respiratory distress syndrome coronavirus 2 (SARS-CoV-2) pandemic, causing coronavirus disease (COVID-19). SARS-CoV-2 is an enveloped RNA virus with cell entry facilitated by spike (S) protein that has a cleavage site at the S1–S2 boundary and other structural proteins such as membrane (M), envelope (E), and nucleocapsid (N) proteins [1]. Currently, there are two lineages of SARS-CoV-2 virus infecting humans, with similar virulence and clinical outcomes, derived from a common ancestor that originated in December 2019 in Wuhan [1, 2]. Most patients who recover from SARS-CoV-2 illness will develop antibodies and memory lymphocytes against these proteins, which gives them immunity [3]. In this editorial, we discuss the biological, operational, and methodological questions that arise when designing a randomised controlled trial (RCT) of convalescent plasma in COVID-19.

## What is convalescent plasma therapy?

Convalescent plasma refers to acellular plasma fraction of blood, containing antibodies against SARS-CoV-2 antigens, with virus neutralisation properties, collected from patients who have recovered from SARS-CoV-2 infections. Passive immunisation with ABO blood group-compatible convalescent plasma will reduce viral burden as neutralising antibodies will binding to the viral spike protein to either prevent interaction with angiotensin-converting enzyme-2 receptor or block the conformational changes in spike protein preventing fusion to host cell membrane and provide immunomodulation.

## What do we know thus far about convalescent plasma therapy in COVID-19 illness?

Since the recent Cochrane review that highlighted very low-certainty evidence on the effectiveness and safety of convalescent plasma in COVID-19 patients [4], Joyner and colleagues have reported safety results from a compassionate use convalescent plasma therapy programme in 5000 adults with COVID-19. They highlight that convalescent plasma is a safe treatment with an overall serious adverse event rate of < 1% (*n* = 36 events), with TACO occuring in 7 patients, TRALI in 11 patients, and allergic transfusion reaction in 3 patients [5]. To date, one RCT has been published. This open-label trial stopped early after recruiting 103 of a planned 200 patients sample size were enrolled. The stoppage was due to low patient recruitment, as the pandemic abated in China, and importantly not for safety reasons [6]. The participants had either severe (respiratory distress and/or hypoxemia) or life-threatening (shock, organ failure, or requiring mechanical ventilation) COVID-19 illness. The intervention, ABO-compatible convalescent plasma at a dose of 4 to 13 ml/kg of recipient body weight, and with an S-RBD-specific IgG titre of at least 1:640. The primary outcome was time to clinical improvement within 28 days, defined as patient discharged alive or reduction of 2 points on a 6-point disease severity scale. The overall trial result was no statistically significant improvement in time to clinical improvement within 28 days between convalescent plasma with standard of care versus standard of care alone. However, any inference from this trial is limited by it's early termination.

## Why do we need more RCTs of convalescent plasma?

The risks of administering plasma screened for common blood-borne pathogens are small, but include allergy/anaphylaxis, transfusion-related acute lung injury (TRALI), and transfusion-associated circulatory overload (TACO) [7]. TRALI and TACO are relevant as many COVID-19 patients have incipient respiratory failure that may worsen with convalescent plasma transfusion-related volume loading. Another specific concern with this intervention is antibody-dependent enhancement (ADE). In SARS-1 coronaviruses, ADE occurs by S protein neutralising antibodies enhancing viral entry into cells though fragment-crystallisable (Fc) receptor expressing cells such as monocytes [8]. This has been shown to worsen lung injury in SARS-1 patients [9]. Non-randomised clinical use (compassionate) will not provide evidence of efficacy, which is an important consideration, as passive immunotherapy was ineffective in severe influenza A [10], and Ebola [11]. The impact of these harms would be difficult to identify outside a well-conducted RCT that collects adverse event data in a standardised way, whilst answering the efficacy question.

## Can we rapidly provide convalescent plasma with neutralising antibodies during a pandemic?

Convalescent plasma can be collected safely from individuals who have recovered from laboratory-confirmed SARS-CoV-2 infection, as neutralising antibody responses begin by 14 days and continue to increase over the next few weeks. Currently, it is uncertain how long these antibodies persist, but in other coronavirus infections, neutralising antibodies may persist at high titres for at least 3 months before declining [12]. Therefore, collection of plasma around 28 days after recovery will provide an effective product with high titres of neutralising antibodies.

However, neither the method to assess viral neutralisation ability of convalescent plasma prior to administration nor the minimum titre of neutralising antibody that is required for treating critically ill patients with COVID-19 is known. There are two methods to assess viral neutralisation ability—pseudotype and live-virus assays. Pseudotype assays using harmless viruses that express the coronavirus spike protein, the target of neutralising antibodies, are a safer, easier, and more sensitive method for detecting neutralising antibody than live-virus assays that assess neutralisation of invasion of tissue culture cells by live virus [13]. The titres of antibody dose vary between studies, from 400 ml of ABO-compatible convalescent plasma with neutralising antibody titre > 1:40 [14] to single 200 ml dose of inactivated convalescent plasma with neutralising antibody titre > 1:640 [15].

## What are the key design issues to consider in RCTs of convalescent plasma?

Current trials include *participants* with a range of COVID-19 illness spectrum, the *intervention* (convalescent plasma different timing, different doses, and need for molecular evidence of viral infection) and *comparators* are different, ranging from standard of care to use of regular plasma for blinding that adds transfusion-related risks in comparator population, and *outcomes* differ between trials. It is conceivable that the treatment effect of convalescent plasma may differ by illness severity, by dose in terms of volume, concentration of neutralisation antibody, and the risk of ADE along with other adverse events during COVID-19 illness (Table 1) [4].

**Table 1:** Ongoing randomised controlled trials of convalescent plasma in COVID-19 illness assessed using the PICO framework. These RCTs were identified in a recent Cochrane review by Valk et al. [4]. *Participants*: We report the setting (severely ill/critically ill versus general wards). In high-risk non-ventilated patients (high inspired oxygen, and/or non-invasive ventilation), this could reduce the need for mechanical ventilation. In ventilated patients, this may translate into improved mortality and reduced length of critical care stay. *Intervention*: For intervention, we report the description of convalescent plasma volume and titres if highlighted. In SARS-1 patients, convalescent plasma improved outcomes when administered within 14 days of illness onset and in those without detectable antibodies against coronavirus at the time of infusion. Only four studies use a predetermined neutralising titre cutoff with convalescent plasma. *Comparator*: We highlight whether the ordinary plasma or standard of care was chosen. In five RCTs, the comparator is ordinary plasma transfusion, which may enhance blinding but comes with risks of blood product. When summarising the ongoing current trials, it is unlikely that an efficacy signal would be generated from many of these trials due their methodological limitations (such as small sample size) and biological limitations (such as lack of pre-defined cutoff for neutralising antibody titres). For outcome, we list only the primary outcome for the trial. We also highlight the proposed sample size in the trial.

Trial ID [country]	Participants	Intervention	Comparator	Outcome	*N*
ChiCTR2000029757 [China]	Severely ill/critically ill	Volume = NR	Standard of care	2-point improvement in clinical symptoms in a 6-point scale	200
		Titres = NR			
ChiCTR2000030010 [China]	Severely ill adults less than 70 years	Volume = NR	Ordinary plasma	2-point improvement in clinical symptoms in a 6-point scale	100
		Titres = NR			
ChiCTR2000030179 [China]	Severely ill adults less than 66 years	Volume = NR	Standard of care	Cure rate	100
		Titres = NR		Mortality	
ChiCTR2000030627 [China]	Severely ill/critically ill	Volume = NR	Standard of care	Temperature control	30
		Titres = NR			
ChiCTR2000030702 [China]	Hospitalised patients	Volume = NR	Standard of care	Time to clinical recovery after randomisation	50
		Titres = NR			
ChiCTR2000030929 [China]	Severely ill adults less than 70 years	Volume = NR	Ordinary plasma	2-point improvement in clinical symptoms in a 6-point scale	60
		Titres = NR			
EUCTR2020-001310-38 [Germany]	Severely ill/critically ill adults less than 75 years	Volume = up to 960 ml	Standard of care	Composite endpoint: - Survival and no longer fulfilling criteria of severe COVID-19 within 21 days after randomisation	120
		Titres = NR			
IRCT20200310046736N1 [Iran]	Adult (20 to 45 years)	Volume = 800 ml	Standard of care	N/A	45
		Titres = NR			
IRCT20200404046948N1 [Iran]	Severely ill/critically ill adults less than 70 years	Volume = up to 500 ml	Standard of care	2-point improvement in clinical symptoms at 14 days	60
		Titres = NR			
IRCT20200409047007N1 [Iran]	Critically ill adults 50–75 years with Pao2/FIO2 ratio < 300; normal IgA level and within 7 days of admission	Volume = up to 500 ml	Standard of care	1-month mortality	35
		Titres = NR			
IRCT20200413047056N1 [Iran]	Severely ill/critically ill adults less than 50 years	Volume = up to 400 ml	Standard of care or intravenous immunoglobulin	NR	15(1:1:1) 3-arm study
		Titres = NR			
NCT04332835 [Columbia]	Hospitalised adults less than 60 years	Volume = up to 500 ml	Hydroxychloroquine	Change in viral load	60
		Titres = NR		Change in antibody titres	
		Coadministration of hydroxychloroquine			
NCT04333251 [USA]	Hospitalised adults	Volume = 2 doses	Standard of care	Reduction in oxygen and ventilation support	115
		Titres = > 1:64			
NCT04342182 [Netherlands]	Hospitalised adults	Volume = up to 300 ml	Standard of care	Mortality	426
		Titres = NR			
NCT04344535 [USA]	Hospitalised adults	Volume = up to 550 ml	Standard plasma	Ventilator-free days up to day 28	500
		Titres = > 1:320			
NCT04345289 [Denmark]	Hospitalised adults with pneumonia	Volume = 600 ml	Multiple interventions; adaptive platform trial	Composite endpoint of all-cause mortality or need of invasive mechanical ventilation up to 28 days	1500
		Titres = NR			1:1:1:1:1:1
NCT04345523 [Spain]	Hospitalised adults with pneumonia	Volume = 800 ml	Standard of care	WHO ordinal scale	278
		Titres = NR			
NCT04345991 [France]	Mild severity as described in the WHO scale, within 8 days	Volume = 800 ml	Standard of care	Survival without needs of ventilator utilisation or use of immunomodulatory drugs at 14 days	120
		Titres = NR			
NCT04346446 [India]	Severely ill/critically ill adults less than 65 years	Volume = up to 600 ml	Standard of care	Proportion of patients remaining free of mechanical ventilation at 7 days	40
		Titres = NR			
NCT04348656 [Canada]	Hospitalised adults receiving supplemental oxygen	Volume = up to 500 ml	Standard of care	Intubation or hospital mortality within 30 days	1200
		Titres = NR			
NCT04355767 [USA]	Adults requiring emergency department evaluation	Volume = up to 600 ml	Standard plasma	Time to disease progression at 15 days	206
		Titres = > 1:80			
NCT04356534 [Bahrain]	Adults > 21 years with severely ill with radiological evidence of pneumonia	Volume = up to 600 ml	Standard of care	Requirement for invasive ventilation	40
		Titres = > 1:80			
NCT02735707 [Multinational]	Severely ill/critically ill adults	Volume = up to 600 ml	Multiple interventions; adaptive platform trial	Days alive and outside of ICU at 21 days	7100 platform
		Titres = > 1:64			

In summary, there is a clear biological framework for considering convalescent plasma as a potential intervention in COVID-19 illness. However, we need high-quality randomised controlled trials prior to using convalescent plasma as standard care in SARS-CoV-2 infections.

## Data Availability

Not applicable.
